# Non-invasive imaging techniques for diagnosis of pelvic deep endometriosis and endometriosis classification systems: an International Consensus Statement^†^^,^^‡^

**DOI:** 10.1093/hropen/hoae029

**Published:** 2024-05-29

**Authors:** G Condous, B Gerges, I Thomassin-Naggara, C Becker, C Tomassetti, H Krentel, B J van Herendael, M Malzoni, M S Abrao, E Saridogan, J Keckstein, G Hudelist, Intersociety Consensus Group §, K Aas-Eng, J L Alcazar, C Bafort, M Bazot, D Bielen, A Bokor, T Bourne, F Carmona, A Di Giovanni, D Djokovic, A Egekvist, J English, C Exacoustos, H Ferreira, S Ferrero, R Forstner, S Freeman, M Goncalves, G Grimbizis, A Guerra, S Guerriero, F W Jansen, D Jurkovic, S Khazali, M Leonardi, C Maciel, L Manganaro, M Mueller, M Nisolle, G Noe, S Reid, H Roman, P Rousset, M Seyer Hansen, S Singh, V Thomas, D Timmerman, U A Ulrich, T Van den Bosch, D Van Schoubroeck, A Wattiez

**Affiliations:** Acute Gynaecology, Early Pregnancy & Advanced Endosurgery Unit, Sydney Medical School Nepean, University of Sydney, Nepean Hospital, Penrith, NSW, Australia; Acute Gynaecology, Early Pregnancy & Advanced Endosurgery Unit, Sydney Medical School Nepean, University of Sydney, Nepean Hospital, Penrith, NSW, Australia; Sydney West Advanced Pelvic Surgery (SWAPS), Blacktown Hospital, Blacktown, NSW, Australia; APHP Hopital Tenon, Department of Radiology, Sorbonne Université, Paris, France; Nuffield Department of Women’s and Reproductive Health, Endometriosis CaRe Centre Oxford, University of Oxford, Oxford, UK; Department of Gynaecology and Obstetrics, University Hospitals Leuven, Leuven, Belgium; Department of Development and Regeneration, Faculty of Medicine, KU Leuven, Leuven, Belgium; Department of Gynecology, Obstetrics and Gynecological Oncology, Bethesda Hospital, Duisburg, Germany; Department of Minimally Invasive Gynecologic Surgery, Ziekenhuis Netwerk Antwerpen Campus Stuivenberg, Antwerp, Belgium; Università degli Studi dell‘Insubria, Varese, Italy; Endoscopica Malzoni, Centre for Advanced Pelvic Surgery, Avellino, Italy; Disciplina de Ginecologia, Departamento de Obstetricia e Ginecologia, Faculdade de Medicina FMUSP, Universidade de Sao Paulo, Sao Paulo, Brazil; Department of Obstetrics and Gynaecology, University College London Hospital, London, UK; Stiftung Endometrioseforschung (SEF), Westerstede, Germany; Department of Gynecology, Center for Endometriosis, Hospital St John of God Vienna, Vienna, Austria; Rudolfinerhaus Private Clinic & Campus, Vienna, Austria

**Keywords:** deep endometriosis, transvaginal ultrasound, MRI, collaboration, non-invasive diagnosis

## Abstract

The International Society of Ultrasound in Obstetrics and Gynecology (ISUOG) and International Deep Endometriosis Analysis (IDEA) group, the European Endometriosis League (EEL), the European Society for Gynaecological Endoscopy (ESGE), ESHRE, the International Society for Gynecologic Endoscopy (ISGE), the American Association of Gynecologic Laparoscopists (AAGL) and the European Society of Urogenital Radiology (ESUR) elected an international, multidisciplinary panel of gynecological surgeons, sonographers, and radiologists, including a steering committee, which searched the literature for relevant articles in order to review the literature and provide evidence-based and clinically relevant statements on the use of imaging techniques for non-invasive diagnosis and classification of pelvic deep endometriosis. Preliminary statements were drafted based on review of the relevant literature. Following two rounds of revisions and voting orchestrated by chairs of the participating societies, consensus statements were finalized. A final version of the document was then resubmitted to the society chairs for approval. Twenty statements were drafted, of which 14 reached strong and three moderate agreement after the first voting round. The remaining three statements were discussed by all members of the steering committee and society chairs and rephrased, followed by an additional round of voting. At the conclusion of the process, 14 statements had strong and five statements moderate agreement, with one statement left in equipoise. This consensus work aims to guide clinicians involved in treating women with suspected endometriosis during patient assessment, counselling, and planning of surgical treatment strategies.

## Introduction

Reducing the diagnostic delay of endometriosis to facilitate adequate action requires a shift from a surgically or lesion-oriented diagnosis to a more comprehensive diagnosis, taking into account not only symptoms and signs, but also non-invasive findings on physical examination and imaging. The latter are contributing increasingly to clinical diagnosis and timely intervention (Agarwal *et al.*, 2019). Various non-invasive imaging techniques have been advocated over the past few decades for non-surgical visualization of pelvic endometriosis. Among these, ultrasound, primarily using a transvaginal approach, is the imaging modality used most commonly for investigation of women with suspected endometriosis, alongside MRI (Bielen *et al.*, 2020) and, less commonly, computed tomography (CT) (Pascoal *et al.*, 2022) or other radiological techniques, such as barium enema and intravenous urography.

It is of pivotal importance for patient counseling and planning of treatment strategies to achieve an accurate diagnosis of endometriosis on imaging, especially deep endometriosis (DE), which is observed in ∼20% of cases of endometriosis (Abrao *et al.*, 2015). Prior to surgery, the diagnosis of DE can be used to predict operative difficulty and, equally important, in the context of infertility, particularly involving ovarian endometriosis, it can assist in the decision regarding whether to treat with surgery or apply assisted reproductive technologies, especially when used in combination with predictive tools, such as the Endometriosis Fertility Index (EFI) (Adamson and Pasta, 2010; Vesali *et al.*, 2020; Tomassetti *et al.*, 2021). The study of Goncalves *et al.* (2021) concluded that systematic evaluation of endometriosis by transvaginal ultrasound (TVS) can accurately replace diagnostic laparoscopy, particularly for DE and ovarian endometriosis. This view is also supported by the recently published updated version of the ESHRE Endometriosis Guideline (Becker *et al.*, 2022), which states that the requirement for histological confirmation for diagnosis of endometriosis is in need of refinement due to ‘…advances in the quality and availability of imaging modalities for at least some forms of endometriosis on the one hand and the operative risk, limited access to highly qualified surgeons and financial implications on the other’.

Ideally, patients with severe DE should be seen at a tertiary referral center, as they may benefit from input from a multidisciplinary team comprising gynecologists, urologists, colorectal surgeons, and specialists in reproductive medicine and imaging (Bendifallah *et al.*, 2018), hence the importance of detailed presurgical characterization and classification of endometriosis, especially DE (Abrao *et al.*, 2015). Several attempts have been made to evaluate the use of current classification and scoring systems incorporating non-invasive imaging techniques in order to facilitate these processes (Hudelist *et al.*, 2021a). However, the environmental impact of non-invasive imaging techniques for endometriosis should be recognized in these times of climate crisis. A recent study by McAlister *et al.* (2022) calculated the carbon footprint of imaging by MRI, CT, and ultrasound in Australia. Of the three modalities, MRI exhibited the largest carbon footprint, followed by CT and then ultrasound. Their impact is attributable mainly to energy consumption and, to some extent, to consumables. Hence, when choosing an imaging technique for patients with suspected endometriosis, physicians should take into consideration that ultrasound has the smallest environmental impact.

The International Society of Ultrasound in Obstetrics and Gynecology (ISUOG) and International Deep Endometriosis Analysis (IDEA) group, the European Society for Gynaecological Endoscopy (ESGE), the European Endometriosis League (EEL), the International Society for Gynecologic Endoscopy (ISGE), ESHRE, the European Society of Urogenital Radiology (ESUR) and the American Association of Gynecologic Laparoscopists (AAGL) therefore formed a working group to develop evidence-based statements to guide the use of non-invasive imaging techniques for diagnosis and classification of pelvic DE, presented in this joint Consensus Statement. Adenomyosis, ovarian endometrioma, superficial and extrapelvic endometriosis, adhesions, biomarkers, economic analysis of these techniques, and pathohistological and/or surgical methods for classification and diagnosis of endometriosis are not considered herein.

## Responsibilities

The following statements derive from a consensus process that included all listed authors and collaborators and representatives from the respective societies, and reflect current evidence-based practice and approaches for the non-invasive diagnosis and classification of endometriosis using imaging techniques. We strongly recommend that clinicians in everyday clinical practice apply independent medical judgement and consider the individual situation and needs of the patient when consulting these statements. All authors listed in this work disclaim any responsibility for its use or application and any clinical decisions deriving from the use of these statements.

## Methods

This Consensus Statement was developed in accordance with a protocol used in a previously published Consensus Statement (Timmerman *et al.*, 2021), and involves societies also represented in that work. Using a six-step protocol chaired and organized by Professors George Condous (G.C.) and Gernot Hudelist (G.H.), an international and multidisciplinary working group was established and orchestrated by chairs of each society, referred to herein as society working-group chairs (G. Condous, ISUOG, IDEA; J. Keckstein, E. Saridogan, ESGE; H. Krentel, G. Hudelist, EEL; C. Becker, C. Tomassetti, ESHRE; B.J. van Herendael, ISGE; M.S. Abrao, M. Malzoni, AAGL; I. Thomassin-Naggara, ESUR). The working group included 53 experts with extensive expertise in the field of diagnosis and/or surgical treatment of endometriosis, reflected by research, clinical expertise, administrative responsibilities, and society leadership positions, and comprised 10 radiologists with a special interest and expertise in MRI and TVS, 12 gynecologists with a special interest and expertise in gynecological ultrasound, 13 gynecologists with extensive experience in surgery for DE and gynecological ultrasound, and 18 gynecologists focused exclusively on surgery for DE.

**Figure 1.**
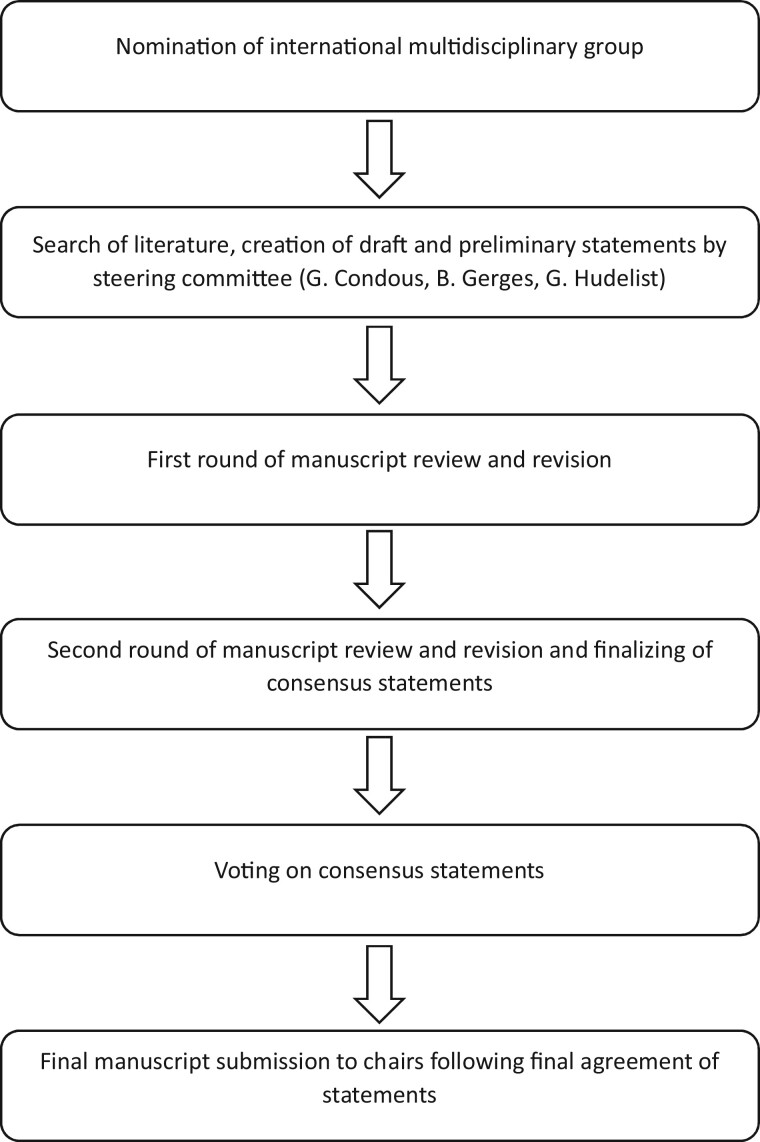
Process for development of Consensus Statement on the use of non-invasive imaging techniques for diagnosis and classification of pelvic deep endometriosis.

A systematic literature review of relevant studies published from inception to February 2023 was carried out by the coordinating chairs (G.C., G.H.) and the joint first author, Bassem Gerges (B.G.), using the MEDLINE, EMBASE, Google Scholar, PubMed, and Scopus databases (Supplementary File S1). The protocol and following methodology, being standard for systematic reviews and meta-analyses, have been described in detail in a previously published study (Gerges *et al.*, 2021a). The literature search was limited to publications in the English language. Editorials, letters, and case reports were excluded, with priority given to systematic reviews, meta-analyses, and validating cohort studies. Additionally, the reference list of each identified article was reviewed for other potentially relevant articles. The coordinating chairs (G.C., G.H.) and joint first author (B.G.) formulated the preliminary consensus statements and were responsible for the first draft of the manuscript. This was followed by distribution of the manuscript to the society chairs, who again distributed and discussed it with all group members, followed by a first round of revisions coordinated by the society chairs. Group members had the opportunity to provide comments and suggestions with their resubmitted versions of the manuscript draft, and statements were modified if there was a lack of consensus among them. The society working-group chairs then submitted the results and comments of the first draft to the coordinating chairs (G.C., G.H.) and joint first author (B.G.) and suggested revisions of the statements if necessary. A revised version of the manuscript was produced and resubmitted to working-group chairs, and thereby all group members, and the process was repeated. Based on the results of the second round, the work and consensus statements were finalized, resulting in 20 statements achieved during this process. Society group members were then able to vote in a binary fashion (agree/disagree), or to abstain from voting in cases of conflict of interest. Statements were classified as having strong agreement (more than 80% of voters agreed), moderate agreement (60%–80% agreed), equipoise (40%–60% agreed), or disagreement (fewer than 40% agreed). A final version of the document was then submitted to all group chairs of the respective societies for approval (Fig. 1). A summary of the supporting evidence, all final consensus statements, and their levels of evidence and grades are presented in Supplementary File S2.

## Literature review

### Transvaginal sonography

#### Rectosigmoid DE

Since Bazot *et al.* (2004) evaluated the accuracy of TVS against surgical findings of pelvic DE, there have been a considerable number of studies published assessing preoperatively imaging techniques to detect DE, in particular rectosigmoid DE. Of these, TVS is the most studied, and is often used as the first-line modality, given its accessibility, relatively low cost and non-invasiveness (Piessens *et al.*, 2014). In the Cochrane review published by Nisenblat *et al.* (2016), the overall pooled sensitivity and specificity for TVS were 90% and 96%, respectively (14 studies). Noventa *et al.* (2019) performed a meta-analysis using a head-to-head approach and, on comparison of TVS versus MRI studies, they found the pooled sensitivity of TVS to be 85% and the specificity, based on their data, was 94%. Subsequently, there were two well-conducted meta-analyses, although they each included a small number of studies, specifically eight (Moura *et al.*, 2019) and 11 (Pereira *et al.*, 2020). Moura *et al.* (2019) performed a meta-analysis comparing TVS and MRI in the diagnosis of rectosigmoid DE in the same population, and found TVS to be marginally superior to MRI, with sensitivities of 90% and 88%, respectively, and specificities of 96% and 90%. Pereira *et al.* (2020) published a comparative study of TVS and MRI, including enhancing techniques, and reported a sensitivity and specificity of 80% and 94%, respectively, for TVS. Most recently, Gerges *et al.* (2021a) performed a systematic review and meta-analysis of prospective studies, limited to those with at least 10 affected and 10 unaffected patients, and found an overall pooled sensitivity of studies assessing TVS for the detection of rectal/rectosigmoid DE (21 studies) of 89%, and specificity of 97%. Furthermore, in their subgroup analyses of 13 studies using 2-dimensional (2D) TVS and five studies using TVS with rectal water contrast (RWC), the sensitivities and specificities were similar, at 84% and 97%, respectively, for 2D-TVS, and 88% and 97%, respectively, for TVS-RWC. A comparison of the included meta-analyses for the detection of rectosigmoid DE is summarized in Table 1.

**Table 1.** Comparison of published meta-analyses on diagnostic accuracy of imaging modalities for detection of deep endometriosis of the rectosigmoid.

Study	Imaging modality	Studies (n)	Patients (n)	Sensitivity	Specificity	LR+	LR−
Hudelist *et al.* (2011)	TVS	10	1106	0.91 (0.88–0.94)	0.98 (0.97–0.99)	30.36 (15.46–59.63)	0.09 (0.05–0.19)
Medeiros *et al.* (2015)	MRI	6	611	0.83 (0.78–0.87)	0.88 (0.84–0.92)	6.92*	0.19*
Guerriero *et al.* (2016b)	TVS	19	2639	0.91 (0.85–0.94)	0.97 (0.95–0.98)	33.6 (17.8–63.5)	0.11 (0.06–0.21)
Nisenblat *et al.* (2016)	TVS	14	1616	0.90 (0.82–0.97)	0.96 (0.94–0.99)	22.50*	0.10*
	MRI	6	612	0.92 (0.86–0.99)	0.96 (0.93–0.98)	23.00*	0.08*
	RES	4	330	0.91 (0.85–0.98)	0.96 (0.91–1.00)	22.75*	0.09*
	CT	3	389	0.98 (0.94–1.00)	0.99 (0.97–1.00)	98.00*	0.02*
	DCBE	2	106	0.56 (0.32–0.80)	0.77 (0.41–1.00)	2.43*	0.57*
Guerriero *et al.* (2018a)	TVS	6	424	0.85 (0.68–0.94)	0.96 (0.85–0.99)	20.4 (4.7–88.5)	0.16 (0.07–0.38)
	MRI	6	424	0.85 (0.78–0.90)	0.95 (0.83–0.99)	18.4 (4.7–72.4)	0.16 (0.11–0.24)
Moura *et al.* (2019)	TVS	8	1132	0.90 (0.87–0.92)	0.96 (0.94–0.97)	20.66 (8.71–49.00)	0.12 (0.08–0.20)
	MRI	8	1132	0.88 (0.85–0.91)	0.90 (0.88–0.92)	17.26 (3.57–83.50)	0.15 (0.10–0.23)
Noventa *et al.* (2019)							
TVS vs MRI	TVS	8	900	0.85 (0.76–0.90)	0.94*	14.17*	0.16*
	MRI	8	900	0.83 (0.76–0.88)	0.93*	11.86*	0.18*
TVS vs RES	TVS	7	710	0.89 (0.84–0.93)	0.95*	17.80*	0.12*
	RES	7	710	0.88 (0.84–0.91)	0.91*	9.78*	0.13*
MRI vs RES	MRI	6	842	0.84 (0.79–0.88)	0.91*	9.33*	0.18*
	RES	6	842	0.91 (0.87–0.94)	0.87*	7.00*	0.10*
Pereira *et al.* (2020)	TVS	11	1362	0.80 (0.62–0.91)	0.94 (0.87–0.97)	13.7 (5.5–34.2)	0.21 (0.10–0.44)
	MRI	11	1362	0.82 (0.68–0.91)	0.94 (0.86–0.97)	13.1 (5.3–32.5)	0.19 (0.10–0.38)
Gerges *et al.* (2021a)	TVS	21	2857	0.89 (0.83–0.92)	0.97 (0.95–0.98)	30.8 (17.6–54.1)	0.12 (0.08–0.17)
	MRI	7	852	0.86 (0.79–0.91)	0.96 (0.94–0.97)	21.0 (13.4–33.1)	0.15 (0.09–0.23)
	CT	6	402	0.93 (0.84–0.97)	0.95 (0.81–0.99)	37.1 (21.1–65.4)	0.08 (0.05–0.14)
	RES	8	850	0.92 (0.87–0.95)	0.98 (0.96–0.99)	20.3 (4.3–94.9)	0.07 (0.03–0.19)

Data in parentheses are 95% CI.

* Value calculated from available study data. CT, computed tomography; DCBE, double contrast barium enema; LR+, positive likelihood ratio; LR−, negative likelihood ratio; RES, transrectal endoscopic sonography; TVS, transvaginal ultrasound.

#### Uterosacral ligaments/torus uterinus, rectovaginal septum and vaginal DE

Despite the uterosacral ligaments (USL) being one of the most commonly affected sites, DE being found at this location during laparoscopy in up to 61% of patients (Fratelli *et al.*, 2013), assessment by TVS of this location is more challenging than at other sites. The performance of TVS for the preoperative diagnosis of USL DE is similar across several published meta-analyses. Nisenblat *et al.* (2016) compared TVS, transrectal sonography, and MRI imaging modalities and found a sensitivity of 64% and specificity of 97% for the detection of USL DE by TVS, from a total of seven studies. Guerriero *et al.* (2015, 2018a) published two reviews: the first, Guerriero *et al.* (2015) assessed TVS and included 11 studies, finding a sensitivity and specificity of 53% and 93%, respectively, while, Guerriero *et al.* (2018a), a head-to-head review comparing TVS and MRI, included six studies and found a sensitivity and specificity for TVS of 67% and 86%, respectively. These results were slightly lower than those of the head-to-head review of Noventa *et al.* (2019) who reported a sensitivity for TVS of 71%, while the specificity calculated from their data was 89%, in the TVS versus MRI analysis, likely due to their inclusion of retrospective studies. The most recent systematic review and meta-analysis, by Gerges *et al.* (2021b), which included prospective studies that assessed preoperatively any imaging modality for the detection of DE in the USL, rectovaginal septum (RVS) and vagina, correlated with the reference standard of surgical data and/or histology, reported a pooled sensitivity and specificity of TVS for USL of 60% and 95%, respectively.

The performance of TVS for the detection of RVS and vaginal DE was found to be poorer than that of other modalities, particularly when compared to MRI. In the first review by Guerriero *et al.* (2015), the sensitivity and specificity of TVS for detection of RVS DE were 49% and 98% and those for vaginal DE were 58% and 96%, respectively. The results were similar for RVS DE in the two head-to-head reviews, with Guerriero *et al.* (2018a) finding a sensitivity and specificity of 59% and 97%, respectively, and Noventa *et al.* (2019) reporting a sensitivity of 47% and with a specificity of 95% calculated from their data. Most recently, Gerges *et al.* (2021b) reported overall pooled sensitivities and specificities of 57% and 100%, respectively, for RVS DE (seven studies) and 52% and 98% for vaginal DE (four studies). A comparison of the included meta-analyses for the detection of USL, RVS, and vaginal DE are summarized in Tables 2, 3, and 4.

**Table 2.** Comparison of published meta-analyses on diagnostic accuracy of imaging modalities for detection of deep endometriosis of the uterosacral ligaments.

Study	Imaging modality	Studies (n)	Patients (n)	Sensitivity	Specificity	LR+	LR−
Guerriero *et al.* (2015)	TVS	11	1482	0.53 (0.35–0.70)	0.93 (0.83–0.97)	7.8 (3.7–16.4)	0.51 (0.36–0.71)
Medeiros *et al.* (2015)	MRI	11	1054	0.85 (0.82–0.88)	0.80 (0.77–0.84)	4.47*	0.19*
Nisenblat *et al.* (2016)	TVS	7	751	0.64 (0.50–0.79)	0.97 (0.93–1.00)	21.33*	0.37*
	MRI	4	199	0.86 (0.80–0.92)	0.84 (0.68–1.00)	5.38*	0.17*
	RES	2	232	0.52 (0.29–0.74)	0.94 (0.86–1.00)	8.67*	0.51*
Guerriero *et al.* (2018a)	TVS	6	261	0.67 (0.55–0.77)	0.86 (0.73–0.93)	4.8 (2.6–9.0)	0.38 (0.29–0.50)
	MRI	6	261	0.70 (0.55–0.82)	0.93 (0.87–0.97)	10.4 (5.1–21.2)	0.32 (0.20–0.51)
Noventa *et al.* (2019)							
TVS vs MRI	TVS	6	636	0.71 (0.65–0.77)	0.89*	6.45*	0.33*
	MRI	6	636	0.67 (0.54–0.77)	0.93*	9.57*	0.35*
TVS vs RES	TVS	5	576	0.75 (0.69–0.70)	0.84*	4.69*	0.30*
	RES	5	576	0.61 (0.43–0.76)	0.69*	1.97*	0.57*
Gerges *et al.* (2021b)	TVS	7	108	0.60 (0.32–0.82)	0.95 (0.90–0.98)	13.2 (8.0–21.8)	0.42 (0.22–0.82)
	MRI	4	440	0.81 (0.66–0.90)	0.83 (0.62–0.94)	4.8 (2.1–11.1)	0.23 (0.14–0.38)

Data in parentheses are 95% CI.

* Value calculated from available study data. LR+, positive likelihood ratio; LR−, negative likelihood ratio; RES, transrectal endoscopic sonography; TVS, transvaginal ultrasound.

**Table 3.** Comparison of published meta-analyses on diagnostic accuracy of imaging modalities for detection of deep endometriosis of the rectovaginal septum.

Study	Imaging modality	Studies (n)	Patients (n)	Sensitivity	Specificity	LR+	LR−
Guerriero *et al.* (2015)	TVS	10	1482	0.49 (0.36–0.62)	0.98 (0.95–0.99)	26.9 (10.2–71.3)	0.52 (0.40–0.67)
Medeiros *et al.* (2015)	MRI	7	753	0.77 (0.69–0.83)	0.95 (0.92–0.96)	15.40*	0.24*
Nisenblat *et al.* (2016)	TVS	10	983	0.88 (0.82–0.94)	1.00 (0.98–1.00)	—^†^	0.12*
	MRI	3	288	0.81 (0.70–0.93)	0.86 (0.78–0.95)	5.79*	0.22*
	RES	2	232	0.78 (0.51–1.00)	0.96 (0.89–1.00)	19.50*	0.23*
Guerriero *et al.* (2018a)	TVS	5	365	0.59 (0.26–0.86)	0.97 (0.94–0.99)	23.5 (9.1–60.5)	0.42 (0.18–0.97)
	MRI	5	365	0.66 (0.51–0.79)	0.97 (0.89–0.99)	22.5 (6.7–76.2)	0.38 (0.23–0.52)
Noventa *et al.* (2019)							
TVS vs MRI	TVS	7	715	0.47 (0.23–0.72)	0.95*	9.40*	0.56*
	MRI	7	715	0.61 (0.48–0.72)	0.92*	7.63*	0.58*
TVS vs RES	TVS	5	574	0.39 (0.13–0.73)	0.95*	7.80*	0.64*
	RES	5	574	0.55 (0.22–0.84)	0.89*	5.00*	0.51*
MRI vs RES	MRI	5	601	0.55 (0.41–0.67)	0.94*	9.17*	0.48*
	RES	5	601	0.55 (0.22–0.84)	0.89*	5.00*	0.51*
Gerges *et al.* (2021b)	TVS	7	1005	0.57 (0.30–0.80)	1.00 (0.92–1.00)	147.1 (7.5–2895.2)	0.44 (0.23–0.81)

Data in parentheses are 95% CI.

* Value calculated from available study data.

† Value could not be calculated from available study data. LR+, positive likelihood ratio; LR−, negative likelihood ratio; RES, transrectal endoscopic sonography; TVS, transvaginal ultrasound.

**Table 4.** Comparison of published meta-analyses on diagnostic accuracy of imaging modalities for detection of deep endometriosis of the vagina.

Study	Imaging modality	Studies (n)	Patients (n)	Sensitivity	Specificity	LR+	LR−
Guerriero *et al.* (2015)	TVS	9	965	0.58 (0.40–0.74)	0.96 (0.87–0.99)	15.3 (4.6–51.3)	0.44 (0.29–0.66)
Medeiros *et al.* (2015)	MRI	9	1021	0.82 (0.76–0.86)	0.82 (0.76–0.86)	4.56*	0.22*
Nisenblat *et al.* (2016)	TVS	6	679	0.57 (0.21–0.94)	0.99 (0.96–1.00)	57.00*	0.43*
	MRI	4	248	0.77 (0.67–0.88)	0.97 (0.92–1.00)	25.67*	0.67*
	RES	2	232	0.39 (0.08–0.70)	1.00 (1.00–1.00)	—^†^	0.61*
Gerges *et al.* (2021b)	TVS	4	451	0.52 (0.29–0.74)	0.98 (0.95–0.99)	27.1 (12.0–61.4)	0.49 (0.30–0.80)
	MRI	3	137	0.64 (0.40–0.83)	0.98 (0.83–0.99)	27.5 (8.4–90.8)	0.37 (0.19–0.69)

Data in parentheses are 95% CI.

* Value calculated from available study data.

† Value could not be calculated from available study data. LR+, positive likelihood ratio; LR−, negative likelihood ratio; RES, transrectal endoscopic sonography; TVS, transvaginal ultrasound.

Since the publication in 2016 of the IDEA consensus opinion (Guerriero *et al.*, 2016a) regarding the sonographic evaluation of the pelvis in women with suspected endometriosis, there has been further delineation of the anatomical terminology used in diagnostic imaging to define the parametrium, paracervix, and USL (Mariani *et al.*, 2021; Scioscia *et al.*, 2021; Di Giovanni *et al.*, 2022). This is of particular significance as parametrial DE can be associated with ureteral stenosis, with associated increased operative risks and the potential need for multidisciplinary surgery. In 2021, Guerriero *et al.* (2021) published a systematic review and meta-analysis of the accuracy of TVS for the detection of parametrial DE, which included four studies. The pooled sensitivity was 31% and the specificity was 98%, although a positive result on TVS significantly increased the post-test probability, from 18% to 79%. More recently, in a retrospective review, Roditis *et al.* (2023) found the sensitivity and specificity for the detection of parametrial DE to be 20.7% and 97.1%, respectively, for TVS, and 36% and 93.8% for MRI.

#### Bladder DE

DE involving the urinary tract, namely the bladder, ureters, and kidneys, is a form of DE affecting between 19% and 53% of women with pelvic DE, but only 1–2% of people affected by endometriosis (Saccardi *et al.*, 2017). Given the low incidence of this manifestation of DE, there are limited systematic reviews assessing the preoperative diagnostic accuracy of imaging for bladder DE. Guerriero *et al.* (2015) performed a systematic review including prospective and retrospective studies that each had at least 50 participants who underwent TVS prior to surgery and found a pooled sensitivity and specificity of 62% and 100%, respectively. Noventa *et al.* (2019) performed a systematic review of head-to-head studies, including retrospective studies, with only two studies that compared TVS and transrectal endoscopic sonography (RES). They found, by univariate analysis, diagnostic odds ratios of 4.94 for TVS and 3.13 for RES. In a review of prospective studies which assessed preoperatively any imaging modality for the presence of bladder DE, correlated with the gold standard of surgical data and/or histology as reference, and with at least 10 affected and 10 unaffected patients, Gerges *et al.* (2021c) found an overall pooled sensitivity for detection of bladder DE of 55% and specificity of 99%, although a meta-analysis could not be performed given the limited number of applicable studies. A comparison of the included meta-analyses for the detection of bladder DE is summarized in Table 5.

**Table 5.** Comparison of published meta-analyses on diagnostic accuracy of imaging modalities for detection of deep endometriosis of the bladder.

Study	Imaging modality	Studies (n)	Patients (n)	Sensitivity	Specificity	LR+	LR−
Guerriero *et al.* (2015)	TVS	8	1248	0.62 (0.40–0.80)	1.00 (0.97–1.00)	208.4 (21.0–2066.0)	0.38 (0.22–0.66)
Medeiros *et al.* (2015)	MRI	5	586	0.64 (0.48–0.77)	0.98 (0.96–0.99)	31.00*	0.37*
Gerges *et al.* (2021c)	TVS	8	1052	0.55 (0.28–0.79)	0.99 (0.98–1.00)	54.5 (18.9–157.4)	0.46 (0.25–0.85)

Data in parentheses are 95% CI.

* Value calculated from available study data. LR+, positive likelihood ratio; LR−, negative likelihood ratio; TVS, transvaginal ultrasound.

### Magnetic resonance imaging

#### Rectosigmoid DE

The 2016 Cochrane review of Nisenblat *et al.* (2016) reported an overall sensitivity and specificity for MRI of 92% and 96%, respectively (six studies). More recently, Noventa *et al.* (2019) performed a meta-analysis using a head-to-head approach and found a pooled sensitivity for MRI of 83%, with a specificity calculated from their data of 93%, when compared with TVS (at 85% and 94%) and 84% and 91%, respectively, when compared with RES (at 91% and 87%). Moura *et al.* (2019) performed a meta-analysis comparing MRI versus TVS in the diagnosis of rectosigmoid DE in the same population. Both modalities were found to have similar sensitivities (88% vs 90%) and specificities (90% vs 96%). Pereira *et al.* (2020) published a comparative study of MRI versus TVS, including enhancing techniques, and reported sensitivities of 82% versus 80% and specificities of 94% versus 94%. However, the latter two meta-analyses (Moura *et al.*, 2019; Pereira *et al.*, 2020), although well conducted, each included a small number of studies: eight and 11, respectively. More recently, Gerges *et al.* (2021a) performed a systematic review and meta-analysis of prospective studies, limited to those with at least 10 affected and 10 unaffected patients, and found the overall pooled sensitivity and specificity of all studies assessing MRI (seven studies, 852 patients) to be 86% and 96%, respectively, while the subgroup analysis of 2D-MRI (five studies, 813 patients) had similar results, with a sensitivity and specificity of 85% and 96%, respectively. Due to the limited number of studies, other subgroup analyses were not performed. In a study assessing interobserver agreement, three-dimensional (3D) MRI performed similarly to 2D-MRI for the detection of rectosigmoid DE, with sensitivities for radiologists interpreting 3D-MRI ranging from 89% to 100% and specificities from 94% to 100% (Bazot *et al.*, 2013), while, in another study, MRI with rectal ultrasound gel outperformed 2D-MRI, with a sensitivity of 99% and specificity of 96%, compared with 85% and 96%, respectively (Hottat *et al.*, 2009). A comparison of the included meta-analyses for the detection of rectosigmoid DE is summarized in Table 1.

#### Uterosacral ligament/torus uterinus, rectovaginal septum and vaginal DE

MRI generally outperforms TVS for the detection of USL DE, especially with respect to sensitivity. Nisenblat *et al.* (2016) compared imaging modalities and found a sensitivity and specificity for the detection of USL DE for MRI (four studies) of 86% and 84%, respectively, compared with 64% and 97% for TVS (seven studies). In the head-to-head review by Guerriero *et al.* (2018a), from a total of six studies, the sensitivity and specificity, respectively, for the detection of USL DE by MRI were 70% and 93%, compared with 67% and 86% for TVS. Similarly, for RVS DE, the sensitivity and specificity for MRI were 66% and 97%, respectively, compared with 59% and 97% for TVS. In contrast, Noventa *et al.* (2019) performed a head-to-head meta-analysis including retrospective studies and found TVS to be slightly superior to MRI for the detection of USL DE, with sensitivities of 71% versus 67% and specificities, based on their data, of 89% versus 93%. In contrast, the reported sensitivities and calculated specificities for the detection of RVS DE were 47% and 95%, respectively, for TVS and 61% and 92% for MRI. In a meta-analysis assessing the performance of MRI in detecting DE, Medeiros *et al.* (2015) reported sensitivities and specificities for USL DE of 85% and 80%, for RVS DE of 77% and 95% and for vaginal DE of 82% and 82%, respectively. Similarly, the meta-analysis of prospective studies by Gerges *et al.* (2021b) found MRI to outperform TVS consistently, with sensitivities and specificities for USL DE of 81% and 83%, respectively, for MRI and 60% and 95% for TVS, and sensitivities and specificities for vaginal DE of 64% and 98%, respectively, for MRI and 52% and 98% for TVS. A comparison of the included meta-analyses for the detection of USL, RVS, and vaginal DE are summarized in Tables 2, 3, and 4.

#### Bladder DE

Studies assessing the diagnostic accuracy of imaging techniques for bladder DE are quite limited in number, largely due to the low incidence of the disease. Medeiros *et al.* (2015) performed a pooled analysis, including both retrospective and prospective studies, of the detection of bladder DE using MRI. They found a pooled sensitivity and specificity of 64% and 98%, respectively. In a review of prospective studies (Gerges *et al.*, 2021c), while pooled analyses could not be performed due to the limited number of studies, two studies were described which assessed 2D-MRI, reporting sensitivities ranging from 50% (Guerriero *et al.*, 2018b) to 100% (Alborzi *et al.*, 2018) and specificities ranging from 97% (Guerriero *et al.*, 2018b) to 100% (Alborzi *et al.*, 2018). MRI with rectal ultrasound gel performed similarly to this, with a sensitivity of 70% and specificity of 100% (Hottat *et al.*, 2009). A comparison of the included meta-analyses for the detection of bladder DE is summarized in Table 5.

### Computed tomography

The use of CT for the preoperative detection of endometriosis is less well-studied compared with TVS and MRI, and mostly it is used for detection of rectosigmoid DE. In the 2021 systematic review by Gerges *et al.* (2021a), six studies were included which assessed CT (402 patients), of which three assessed standard CT (Biscaldi *et al.*, 2007; Ferrero *et al.*, 2011; Stabile Ianora *et al.*, 2013) and three assessed CT colonography (Baggio *et al.*, 2016; Ferrero *et al.*, 2017; Barra *et al.*, 2020). The overall pooled sensitivity and specificity of CT for the detection of rectosigmoid DE were 93% and 95%, respectively (Gerges *et al.*, 2021a). Subanalyses of CT colonography were not performed, and these results ranged widely, with one study (Baggio *et al.*, 2016) finding poor performance, with a sensitivity of 68% and specificity of 67%, while the other two studies reported sensitivities of 93% (Ferrero *et al.*, 2017) and 95% (Barra *et al.*, 2020) and specificities of 87% (Ferrero *et al.*, 2017) and 93% (Barra *et al.*, 2020). The review by Nisenblat *et al.* (2016) reported better results when CT was combined with water enema, with three studies (389 patients) (Ferrero *et al.*, 2011; Stabile Ianora *et al.*, 2013; Baggio *et al.*, 2016) included, resulting in a pooled sensitivity and specificity of 98% and 99%, respectively. However, Nisenblat *et al.* (2016) stated that this technique should be avoided in young patients whenever possible, due to the associated radiation exposure (Biscaldi *et al.*, 2021). This is consistent with the ALARA principle, i.e. ensuring that the exposure to radiation is ‘as low as reasonably achievable’ (Hendee and Edwards, 1986).

### General remarks on imaging

The test performance of any imaging technique is operator-dependent and will increase with increasing levels of training, skills, and experience of the operator. Also, as systematic reviews, by definition, include older studies, and because expertise in imaging of endometriosis has improved dramatically worldwide in the last few years, it is reasonable to assume that the published sensitivity figures are an underestimation of the current status. The following statements should be interpreted based on these assumptions. Also, while, herein, these imaging techniques have been compared with each other in various anatomical areas, they can be complementary and do not need to be used exclusively (Bielen *et al.*, 2020). For example, a recent analysis of the combined use of vaginal palpation, TVS, and MRI found that at least two positive tests were the most valid model for diagnosing DE, with an accuracy of 91.4% (Roditis *et al.*, 2023).

### Non-invasive use of classification and scoring systems for endometriosis

A multitude of classification and scoring systems for topographical description and expression of the extent of endometriosis and associated secondary adhesions have been proposed and in use for decades, with varying rates of recognition among clinicians, radiologists, sonographers, and gynecological surgeons (International working group of AAGL *et al.*, 2021). These include the #Enzian, AAGL classification, EFI, deep Pelvic Endometriosis Index (dPEI), revised American Society of Reproductive Medicine (rASRM) score and Ultrasound-Based Endometriosis Staging System (UBESS).

#### TVS for description and classification of DE

Terms and definitions for uniform description of DE with ultrasound standardized across different centers and countries have been proposed by the IDEA group and are now widely accepted (Guerriero *et al.*, 2016a). These definitions serve primarily as standardized terminology for describing DE with ultrasound. Their use, applicability, accuracy, and reproducibility are currently under investigation in an international multicenter study (IDEA Phase 1). As part of this, Leonardi *et al.* (2022) recently published the results of a pilot study on the accuracy of the IDEA terms and definitions for presurgical detection of DE. This included 273 women with suspected endometriosis, of whom 256 (93.8%) had endometriosis confirmed, of which 190 (74.2%) were DE cases. In these women, the diagnostic accuracy of TVS using IDEA definitions was 86.1%, sensitivity was 88.4%, specificity was 78.8%, positive predictive value (PPV) was 92.9%, negative predictive value (NPV) was 68.4%, positive likelihood ratio (LR+) was 4.17, and negative likelihood ratio (LR−) was 0.15. Applying the IDEA criteria in 537 women with suspected endometriosis, Szabo *et al.* (2024) demonstrated a diagnostic accuracy for TVS in the diagnosis of colorectal DE of 94%, sensitivity of 93.5%, specificity of 94.6%, NPV of 93.1%, PPV of 94.9%, LR+ of 17.24, and LR− of 0.07.

Among all scoring and/or classification systems for endometriosis published so far, the rASRM score (1997) (Supplementary Fig. S1), the #Enzian classification (Keckstein *et al.*, 2003; Keckstein *et al.*, 2021) (Supplementary Fig. S2), the UBESS (Menakaya *et al.*, 2016) (Supplementary Fig. S3), the EFI for prediction of conception following surgery for endometriosis (Adamson and Pasta, 2010; Tomassetti *et al.*, 2021) (Supplementary Fig. S4) and the AAGL endometriosis classification (Abrao *et al.*, 2021) have also been investigated for their non-invasive applicability using TVS and/or MRI. Ideally, it should be possible to describe endometriosis via scoring and classification systems common to all, including surgeons, radiologists, and sonographers, to facilitate communication and clinical research.

The rASRM score defines degrees of severity of endometriosis in four stages (minimal (Stage I), mild (Stage II), moderate (Stage III), and severe (Stage IV)), based on endometriotic lesions affecting the pelvic peritoneum, ovaries, and associated adhesions. Points are allocated according to whether the lesion is deep or superficial, the lesion size, and the type (filmy or dense) and extent of adhesions involving the Fallopian tubes, ovaries, and pouch of Douglas, and are combined to give a total score that corresponds to one of the four possible stages. Leonardi *et al.* (2020) investigated retrospectively the accuracy of TVS for staging endometriosis preoperatively in 204 patients using the rASRM classification. When evaluating the stages separately, the sensitivity, specificity, PPV, and NPV of TVS were 18.2%, 94.7%, 80.0%, and 49.7%, respectively, for rASRM Stage I; 22.7%, 96.7%, 45.5%, and 91.2% for Stage II; 62.5%, 92.0%, 40.0%, and 96.7% for Stage III; and 71.9%, 97.1%, 82.1%, and 94.9% for Stage IV. Similar to this observation of Leonardi *et al.* (2020) that TVS had lower accuracy on assessment in minimal and mild rASRM stages of disease, Holland *et al.* (2010) found low sensitivity of TVS for diagnosing minimal and mild endometriosis but an accuracy of 94% for detection of moderate and severe disease. Of note, both authors observed low diagnostic accuracy for TVS in the detailed assessment of DE, due to the fact that DE could not be scored clearly using the rASRM classification. Finally, Tomassetti *et al.* (2021) found good agreement with findings at laparoscopy using TVS for estimating the EFI, which is based partly on the rASRM. So far, there have been no attempts to use MRI in combination with the rASRM score to describe and diagnose endometriosis.

To improve classification of DE, the Enzian system was developed in 2003 (Keckstein *et al.*, 2003) and further extended and modified in 2021 (Keckstein *et al.*, 2021). Five studies have evaluated the accuracy of TVS in combination with the Enzian classification. Hudelist *et al.* (2021b) compared TVS findings with surgical findings in 195 women with DE and found good agreement between these modalities, especially for Enzian compartments A (vagina, rectovaginal space, retrocervical area), C (rectum), and FB (urinary bladder). TVS detected DE in compartments A, B (USL, cardinal ligaments, pelvic sidewall), C, and FB with sensitivities of 84%, 91%, 92%, and 88%, respectively, and specificities of 85%, 73%, 95%, and 99%. Recently, Enzelsberger *et al.* (2022) evaluated preoperative use of the Enzian classification using TVS and/or MRI in a prospective multicenter study including 1062 women undergoing surgery for endometriosis, and observed lower accuracy, compared with laparoscopic evaluation, for TVS and/or MRI for Enzian compartments A, B, and C. Complete concordance between compartment and imaging Grade 1, 2, or 3 was observed in 369 women (35.14% of 1050 valid ratings), which increased to 40.3% when the numerical ratings in compartments A/B/C were categorized into ‘affected’ (combining Grades 1, 2, and 3) and ‘not affected’ (coded as 0). Overall concordance, sensitivity, specificity, PPV, and NPV, respectively, of TVS and/or MRI relative to surgical evaluation for compartment A were 83%, 63%, 91%, 72%, and 88%, for compartment B were 69%, 47%, 86%, 72%, and 68%, and for compartment C were 89%, 52%, 96%, 76%, and 91%. However, either MRI or TVS could be applied and, also, TVS was performed by sonographers with limited experience in scanning DE, which limits the conclusions that can be drawn from these results regarding the accuracy of TVS when used in combination with the Enzian classification.

##### #Enzian

In order to test the accuracy of the modified Enzian classification, the so-called #Enzian classification, which also takes into account peritoneal and ovarian endometriosis and secondary tubal adhesions, and has been shown to outperform the rASRM score regarding description of the extent of DE (Montanari *et al.*, 2022a), Di Giovanni *et al.* (2023) investigated retrospectively using the #Enzian classification 93 patients who had undergone TVS prior to surgery. They found sensitivities and specificities of TVS in the identification of endometriosis in compartment O (ovary) of 100% and 100%, respectively (right) and 100% and 96% (left), compartment A of 97% and 86%, compartment B of 100% and 90% (right) and 97% and 70% (left), compartment C of 100% and 96%, compartment FB of 86% and 100%, compartment FI (intestinum) of 100% and 100%, and compartment FU (ureters) of 100% and 100%. Bindra *et al.* (2023) reviewed retrospectively 50 patients undergoing surgery following TVS mapping used with #Enzian, and observed accuracy values similar to those reported by Di Giovanni *et al.* (2023). Recently, Montanari *et al.* (2022b) evaluated the #Enzian classification in a prospective, multicenter study, including 745 patients undergoing TVS and surgery for DE. The sensitivity for detection of endometriotic lesions ranged from 50% (#Enzian compartment FI) to 95% (#Enzian A) and specificity ranged from 86% (#Enzian T (tubo-ovarian condition), left) to 99% (#Enzian FI) or 100% (#Enzian FB (urinary bladder), #Enzian FU and #Enzian FO (other extragenital locations)), with PPVs ranging from 90% (#Enzian T, right) to 100% (#Enzian FO), NPVs ranging from 74% (#Enzian B, left) to 99% (#Enzian FB and #Enzian FU) and accuracy ranging from 88% (#Enzian B, right) to 99% (#Enzian FB), confirming that the presence and extent of DE can be evaluated accurately using TVS in combination with the #Enzian classification.

##### UBESS

The UBESS was created in order to stage disease extent and predict the complexity of surgery in patients with DE, based on the anatomical location of DE and sonographic markers of local invasiveness (Menakaya *et al.*, 2016). In a multicenter prospective and retrospective cohort study including 192 consecutive women with suspected endometriosis, three stages of UBESS (I–III) were correlated with three levels of complexity of laparoscopic surgery. The accuracy of UBESS Stage III in predicting the need for advanced laparoscopic surgery was 95.3%, sensitivity was 94.8%, specificity was 95.5%, PPV was 90.2%, NPV was 97.7%, LR+ was 21.2, and LR− was 0.054 (Menakaya *et al.*, 2016). External validation of the UBESS showed it to have little predictive value for surgical difficulty in a small proportion of 33 patients (Chaabane *et al.*, 2019) and revealed problems with generalizability to cases lacking bowel DE or lacking obliteration of the pouch of Douglas (Espada *et al.*, 2021).

##### AAGL classification and EFI

Among other systems for classification and scoring of endometriosis that have been proposed (Vermeulen *et al.*, 2021) is the ultrasound-based 2021 AAGL endometriosis classification (Abrao *et al.*, 2021). This system was evaluated recently by Abrao *et al.* (2023), who showed that it is only accurate in AAGL Stages I and IV and distinguishes reliably AAGL Stages I–II from Stages III–IV. They found that ultrasound best identified endometriosis of the ovaries, bladder, and bowel, but was more limited for the Fallopian tubes and superficial peritoneum. The EFI works primarily as a model to predict fertility outcome following surgery for endometriosis. It constitutes a 10-point scoring system based on factors such as patient characteristics (age, duration of infertility, and history of prior pregnancy), the rASRM classification, and functionality of Fallopian tubes and ovaries during surgery. One study has demonstrated the possibility of applying the EFI with ultrasound instead of invasive methods, showing that the prediction model can be assessed using TVS-based tubal patency testing, with a 10% loss of accuracy compared with the invasive application of EFI (Tomassetti *et al*., 2021).

#### MRI for description and classification of DE

Two consensus MRI lexicons from the Society of Abdominal Radiology (SAR) (Jha *et al.*, 2020) and from the French Society of Women’s Imaging (SIFEM) (Rousset *et al.*, 2023) were published recently. They both describe the different locations of DE according to a compartment-based approach of the pelvis. The most recent one (Rousset *et al.*, 2023) emphasized the description of lateral compartments, which are usually difficult to detect with TVS and are crucial for surgical planning.

Several studies have investigated use of the Enzian classification in conjunction with MRI, reporting good agreement rates between radiological and surgical findings except for B-compartment lesions (Di Paola *et al.*, 2015; Burla *et al.*, 2019; Widschwendter *et al.*, 2022; Fendal Tunca *et al.*, 2023). Manganaro *et al.* (2021) and Burla *et al.* (2021) showed that the Enzian classification based on MRI findings is also reproducible. In addition, Thomassin-Naggara *et al.* (2020) demonstrated that, for DE lesions in compartments A and C, using MRI in conjunction with Enzian classification was accurate in predicting operating time, hospital stay, and postoperative complications according to the Clavien–Dindo classification. However, they highlighted the poor reproducibility of the description of B-compartment lesions due to the difficulty of measuring USL on MRI. The same limitation was noted in a recent prospective international multicenter study performed in 12 centers (1062 women) (Enzelsberger *et al.*, 2022), which demonstrated that MRI-based and surgical Enzian classifications were concordant for DE lesions in compartment A in 78.7% (118/150) of cases and compartment C in 82.7% (124/150) of cases, but only in 34.7% (52/150) of cases with lesions in compartment B. Another MRI classification was published in 2020 (Thomassin-Naggara *et al.*, 2020), the dPEI classification, which demonstrated high reproducibility (kappa = 0.74), including for the USL (Supplementary Fig. S5). This MRI classification includes description of lateral compartments and predicts accurately operating time, hospital stay, and postoperative complications (Thomassin-Naggara *et al.*, 2023). Larger prospective European and American validation studies on the use of MRI-based #Enzian and dPEI classifications are ongoing.

## Consensus statements

### General statements

- The test performance of any imaging technique for the detection of DE is operator-dependent and will increase with exposure, level of training and skills, and experience of the operator.
  Consensus: yes, 96.2% (n=51); no, 0% (n=0); abstain, 3.8% (n=2)
- Patients with a plan for surgical intervention for endometriosis should undergo preoperative imaging for the detection of DE performed by adequately trained operators.
  Consensus: yes, 96.2% (n=51); no, 0% (n=0); abstain, 3.8% (n=2)
- TVS performed by adequately trained operators is recommended as the first-line imaging tool due to its availability, good test performance, cost efficacy, and its low environmental impact when compared to other imaging methods.
  Level of evidence: 1a
  Grade of statement: A
  Consensus: yes, 96.2% (n=51); no, 0% (n=0); abstain, 3.8% (n=2)

### Statements on ultrasonography

- Imaging with TVS can reliably preoperatively predict, and is recommended to detect, the presence of DE of the rectum, but is less accurate in predicting sigmoidal DE due to limited visibility.
  Level of evidence: 1a
  Grade of statement: A
  Consensus: yes, 86.8% (n=46); no, 5.7% (n=3); abstain, 7.5% (n=4)
- Imaging with TVS can help to preoperatively predict the presence of DE of the RVS.
  Level of evidence: 1a
  Grade of statement: B
  Consensus: yes, 83.0% (n=44); no, 3.8% (n=2); abstain, 13.2% (n=7)
- Imaging with TVS can help to preoperatively predict the presence of DE of the vagina, USL, and parametrium.
  Level of evidence: 1a
  Grade of statement: B
  Consensus: yes, 73.6% (n=39); no, 18.9% (n=10); abstain, 7.5% (n=4)
- Imaging with TVS can help to preoperatively predict the presence of DE of the bladder.
  Level of evidence: 1a
  Grade of statement: B
  Consensus: yes, 90.6% (n=48); no, 1.9% (n=1); abstain, 7.5% (n=4)

### Statements on MRI and CT

- Imaging with MRI can reliably preoperatively predict the presence of DE of the rectosigmoid.
  Level of evidence: 1a
  Grade of statement: A
  Consensus: yes, 90.6% (n=48); no, 5.7% (n=3); abstain, 3.8% (n=2)
- Imaging with MRI can reliably preoperatively predict the presence of DE of the USL and torus uterinus.
  Level of evidence: 1a
  Grade of statement: B
  Consensus: yes, 88.7% (n=47); no, 0% (n=0); abstain, 11.3% (n=6)
- Imaging with MRI is helpful to preoperatively predict the presence of DE of the RVS.
  Level of evidence: 1a
  Grade of statement: B
  Consensus: yes, 90.6% (n=48); no, 3.8% (n=2); abstain, 5.7% (n=3)
- Imaging with MRI can reliably preoperatively predict the presence of DE of the vagina.
  Level of evidence: 1a
  Grade of statement: B
  Consensus: yes, 86.8% (n=46); no, 3.8% (n=2); abstain, 9.4% (n=5)
- Imaging with MRI can reliably preoperatively predict the presence of DE of the bladder.
  Level of evidence: 1a
  Grade of statement: B
  Consensus: yes, 92.5% (n=49); no, 3.8% (n=2); abstain, 3.8% (n=2)
- Imaging with CT may reliably preoperatively predict the presence of DE of the rectosigmoid but is less studied than other imaging modalities. There are, however, no obvious advantages compared to MRI, as well as the disadvantage of radiation exposure.
  Level of evidence: 2a
  Grade of statement: B
  Consensus: yes, 69.8% (n=37); no, 22.6% (n=12); abstain, 7.5% (n=4)
- There is insufficient evidence to support, compared to other imaging modalities, the use of CT for the detection of DE of the USL, torus uterinus, RVS, vagina, or bladder.
  Level of evidence: 2a
  Grade of statement: D
  Consensus: yes, 90.6% (n=48); no, 1.9% (n=1); abstain, 7.5% (n=4)

### Statements on the non-invasive use of classification systems

- Imaging with TVS in combination with the rASRM score can help to describe moderate to severe endometriosis, but is less accurate in cases of minimal to mild disease as classified with the rASRM score.
  Level of evidence: 4
  Grade of statement: D
  Consensus: yes, 62.3% (n=33); no, 7.5% (n=4); abstain, 30.2% (n=16)
- Imaging with TVS in combination with the #Enzian classification can reliably describe DE, ovarian endometriosis, and adhesions, but is less accurate in cases of parametrial involvement (compartment B).
  Level of evidence: 1a
  Grade of statement: B
  Consensus: yes, 83.0% (n=44); no, 3.8% (n=2); abstain, 13.2% (n=7)
- Imaging with MRI in combination with the #Enzian classification can reliably describe rectal and RVS DE and ovarian endometriosis, but is less accurate in cases of USL and/or parametrial involvement (compartment B) and adhesions.
  Level of evidence: 4
  Grade of statement: B
  Consensus: yes, 81.1% (n=43); no, 5.7% (n=3); abstain, 13.2% (n=7)
- Imaging with TVS in combination with the UBESS classification may help to estimate surgical complexity, but the predictive value is not yet generalizable.
  Level of evidence: 3b
  Grade of statement: B
  Consensus: yes, 64.2% (n=34); no, 5.7% (n=3); abstain, 30.2% (n=16)
- Imaging alone with TVS and in combination with the EFI prediction cannot be used reliably as a substitute for the EFI generated by invasive, i.e. surgical, methods.
  Level of evidence: 4
  Grade of statement: D
  Consensus: yes, 62.3% (n=33); no, 7.5% (n=4); abstain, 30.2% (n=16)
- Imaging alone with TVS in combination with the AAGL classification may be used as a substitute for the AAGL classification generated by invasive, i.e. surgical, methods.
  Level of evidence: 2b
  Grade of statement: C
  Consensus: yes, 50.9% (n=27); no, 28.3% (n=15); abstain, 20.8% (n=11)

## Discussion

The present work represents a Consensus Statement regarding the use of non-invasive imaging methods, particularly TVS and MRI, in the application of classification systems for the detection of DE. The test performance of any imaging technique is operator-dependent. Imaging with TVS and MRI needs to be performed by well-trained medical staff. TVS is recommended as a first-line imaging tool, due to its availability, good test performance, cost efficacy and low environmental impact. However, it is acknowledged that many centers adopt MRI as a first-line technique, which is also appropriate.

There was strong agreement that TVS assessment of patients with suspected DE will determine accurately or rule out the presence of DE affecting the rectum, RVS, and bladder, but that TVS is less precise in locations such as the parametrium and the USL. However, the detection of DE of the USL and parametrium using TVS is evolving and constantly improving. MRI-based imaging is capable of detecting DE in these locations and a consensus was reached that MRI can reliably predict the presence of USL, parametrial, and RVS DE.

The use of classification systems for DE is a matter of ongoing debate. There was moderate agreement regarding the non-invasive use of rASRM and UBESS classification systems and the EFI prediction model, and equipoise regarding the usefulness of TVS-based use of the AAGL classification. The majority of participants agreed strongly on the use of TVS or MRI in combination with the #Enzian classification, although it is less accurate in cases of parametrial and USL involvement. Future studies on rASRM, AAGL, UBESS, EFI, and #Enzian classification will hopefully further clarify their role in the setting of parametrial and USL involvement.

It is noteworthy that the reference standards in many published studies were laparoscopy, with or without histopathology. Hence, it is difficult to ascertain the limitation of operator expertise, or a reference standard which could be used in women who are managed conservatively. While this Statement focused on non-invasive imaging primarily for planning surgery, this is not the only aspect of endometriosis treatment, because at least 40% of women with DE are asymptomatic. Furthermore, in those with symptoms, it is not always clear that these are caused by or coincide with endometriosis. The statements herein pertain primarily to women with symptomatic disease with a possible plan for surgical treatment. Assessment of women with potential DE by means of non-invasive imaging with TVS and/or MRI performed by appropriately trained clinicians, combined with planning of surgical and/or conservative management approaches, should be the standard of care in healthcare facilities offering endometriosis therapy.

## Citation

This Consensus Statement should be cited as: ‘Condous G, Gerges B, Thomassin-Naggara I, Becker C, Tomassetti C, Krentel H, van Herendael BJ, Malzoni M, Abrao MS, Saridogan E, Keckstein J, Hudelist G, and Intersociety Consensus Group’. Non-invasive imaging techniques for diagnosis of pelvic deep endometriosis and endometriosis classification systems: an International Consensus Statement. *Human Reproduction Open* 2024; **2024**: hoae029. DOI: https://doi.org/10.1093/hropen/hoae029

## Supplementary Material

hoae029_Supplementary_Figures_S1-S5

hoae029_Supplementary_File_S1_S2

## Data Availability

The data underlying this article are available in the article and in its online Supplementary Material.

## Appendix

### Intersociety consensus group:

K. Aas-Eng, *Department of Gynaecology, Oslo University Hospital, Oslo, Norway; Institute of Clinical Medicine, University of Oslo, Oslo, Norway*

J. L. Alcazar, *Department of Obstetrics and Gynecology, Clinica Universitaria de Navarra, University of Navarra School of Medicine, Pamplona, Spain*

C. Bafort, *Department of Gynaecology & Obstetrics, University Hospitals Leuven, Leuven, Belgium; KU Leuven, Faculty of Medicine, Department of Development and Regeneration, Leuven, Belgium*

M. Bazot, *Department of Radiology, Tenon University Hospital, Assistance Publique des Hôpitaux de Paris (AP-HP), Sorbonne Université, Paris, France; Groupe de Recherche Clinique (GRC-6), Centre Expert en Endométriose (C3E), Assistance Publique des Hôpitaux de Paris, Tenon University Hospital, Sorbonne Université, Paris, France*

D. Bielen, *Department of Radiology, University Hospitals, Leuven, Belgium; Faculty of Medicine, Department of Imaging and Pathology, KU Leuven, Leuven, Belgium*

A. Bokor, *Department of Obstetrics and Gynecology, Semmelweis University, Budapest, Hungary*

T. Bourne, *Obstetrics and Gynaecology Unit, Queen Charlotte’s and Chelsea Hospital, Imperial College, London, UK*

F. Carmona, *Department of Gynecology, Institut Clinic of Gynecology, Obstetrics and Neonatology, Hospital Clinic, Institut d'Investigacions Biomèdiques August Pi i Sunyer (IDIBAPS), Barcelona, Spain*

A. Di Giovanni, *Endoscopica Malzoni, Centre for Advanced Pelvic Surgery, Avellino, Italy*

D. Djokovic, *Maternidade Dr Alfredo da Costa, Centro Hospitalar Universitário Lisboa Central, Lisbon, Portugal; NOVA Medical School—Faculdade de Ciências Médicas, NOVA University of Lisbon, Lisbon, Portugal; Hospital CUF Descobertas, Lisbon, Portugal*

A. Egekvist, *Department of Gynaecology and Obstetrics, Aarhus University Hospital, Aarhus, Denmark; Department of Clinical Medicine, Aarhus University, Aarhus, Denmark*

J. English, *Department of Gynaecology, Haaglanden Medisch Centrum, Den Haag, The Netherlands*

C. Exacoustos, *Department of Surgical Sciences, Obstetric/Gynecological Unit, University of Rome ‘Tor Vergata’, Rome, Italy*

H. Ferreira, *Department of Minimally Invasive Surgery Unit, Centro Hospitalar Universitário do Porto, Porto, Portugal*

S. Ferrero, *IRCCS Ospedale Policlinico San Martino, University of Genova, Genova, Italy*

R. Forstner, *Paracelsus Medical University/Uniklinikum Salzburg, Department of Radiology, Salzburg, Austria*

S. Freeman, *Cambridge University Hospitals NHS Foundation Trust, Department of Radiology, Cambridge, UK*

M. Goncalves, *Gynecologic Division, BP—A Beneficencia Portuguesa de Sao Paulo, Sao Paulo, SP, Brazil; Disciplina de Ginecologia, Departamento de Obstetricia e Ginecologia, Faculdade de Medicina FMUSP, Universidade de Sao Paulo, Sao Paulo, SP, Brazil*

G. Grimbizis, *Medical School, Aristotle University of Thessaloniki (Dr Grimbizis), 1st Dept Obstet Gynecol, Thessaloniki, Greece*

A. Guerra, *Hospedal de Luz, Department of Radiology, Lisbon, Portugal*

S. Guerriero, *Centro Integrato di Procreazione Medicalmente Assistita (PMA) e Diagnostica Ostetrico-Ginecologica, Blocco Q, Azienda Ospedaliero Universitaria-Policlinico Duilio Casula, Monserrato, Cagliari, Italy*

F. W. Jansen, *Department of Gynaecology, Leiden University Medical Center, Leiden, The Netherlands*

D. Jurkovic, *Institute for Women's Health, University College Hospital, London, UK*

S. Khazali, *HCA the Lister Hospital—Centre for Endometriosis and Minimally Invasive Gynaecology (CEMIG London), London, UK*

M. Leonardi, *Department of Obstetrics and Gynecology, McMaster University, Hamilton, ON, Canada*

C. Maciel, *Faculdade de Medicina da Universidade do Porto/Centro Hospitalar Universitário de São João Serviço de Radiologia, Porto, Portugal*

L. Manganaro, *Department of Radiological, Oncological and Pathological Sciences, Policlinico Umberto, Sapienza University of Rome, Rome, Italy*

M. Mueller, *Department of Obstetrics and Gynecology, Inselspital, University of Bern, Bern, Switzerland*

M. Nisolle, *Department of Obstetrics and Gynecology, Hospital CHR Liège, University of Liège, Liège, Belgium*

G. Noe, *University of Witten Herdecke and Rheinlandclinics Dormagen, Dormagen, Germany*

S. Reid, *Department of Obstetrics and Gynaecology, Western Sydney University, Sydney, NSW, Australia*

H. Roman, *Franco-European Multidisciplinary Institute of Endometriosis (IFEMEndo), Clinique Tivoli-Ducos, Bordeaux, France*

P. Rousset, *Hospices Civils de Lyon, Department of Radiology, Université Lyon Sud, Lyon, France*

M. Seyer Hansen, *Department of Gynaecology and Obstetrics, Aarhus University Hospital, Aarhus, Denmark; Department of Clinical Medicine, Aarhus University, Aarhus, Denmark*

S. Singh, *Department of Obstetrics and Gynecology, The Ottawa Hospital, Ottawa, ON, Canada*

V. Thomas, *Department of Obstetrics and Gynecology, Tygerberg Hospital, University of Stellenbosch, Cape Town, South Africa*

D. Timmerman, *Department of Development and Regeneration, KU Leuven, Leuven, Belgium; Department of Obstetrics and Gynecology, University Hospitals Leuven, Leuven, Belgium*

U. A. Ulrich, *Department of Obstetrics and Gynecology, Martin Luther Hospital, Berlin, Germany*

T. Van den Bosch, *Department of Obstetrics and Gynaecology, University Hospital Leuven, Leuven, Belgium*

D. Van Schoubroeck, *Department of Obstetrics and Gynecology, University Hospitals, KU Leuven, Leuven, Belgium; Department of Obstetrics and Gynecology, Tienen Regional Hospital, Tienen, Belgium*

A. Wattiez, *Department of Obstetrics and Gynaecology, University of Strasbourg, Strasbourg, France*
