# Supplementary material for: Non-invasive imaging techniques for diagnosis of pelvic deep endometriosis and endometriosis classification systems: an International Consensus Statement,
Source: Hum Reprod Open. 2024 May 29;2024(3):hoae029. doi: 10.1093/hropen/hoae029 (PMC11134890; doi:10.1093/hropen/hoae029)
Supplement: hoae029_Supplementary_Figures_S1-S5 [file hoae029_supplementary_figures_s1-s5.docx]

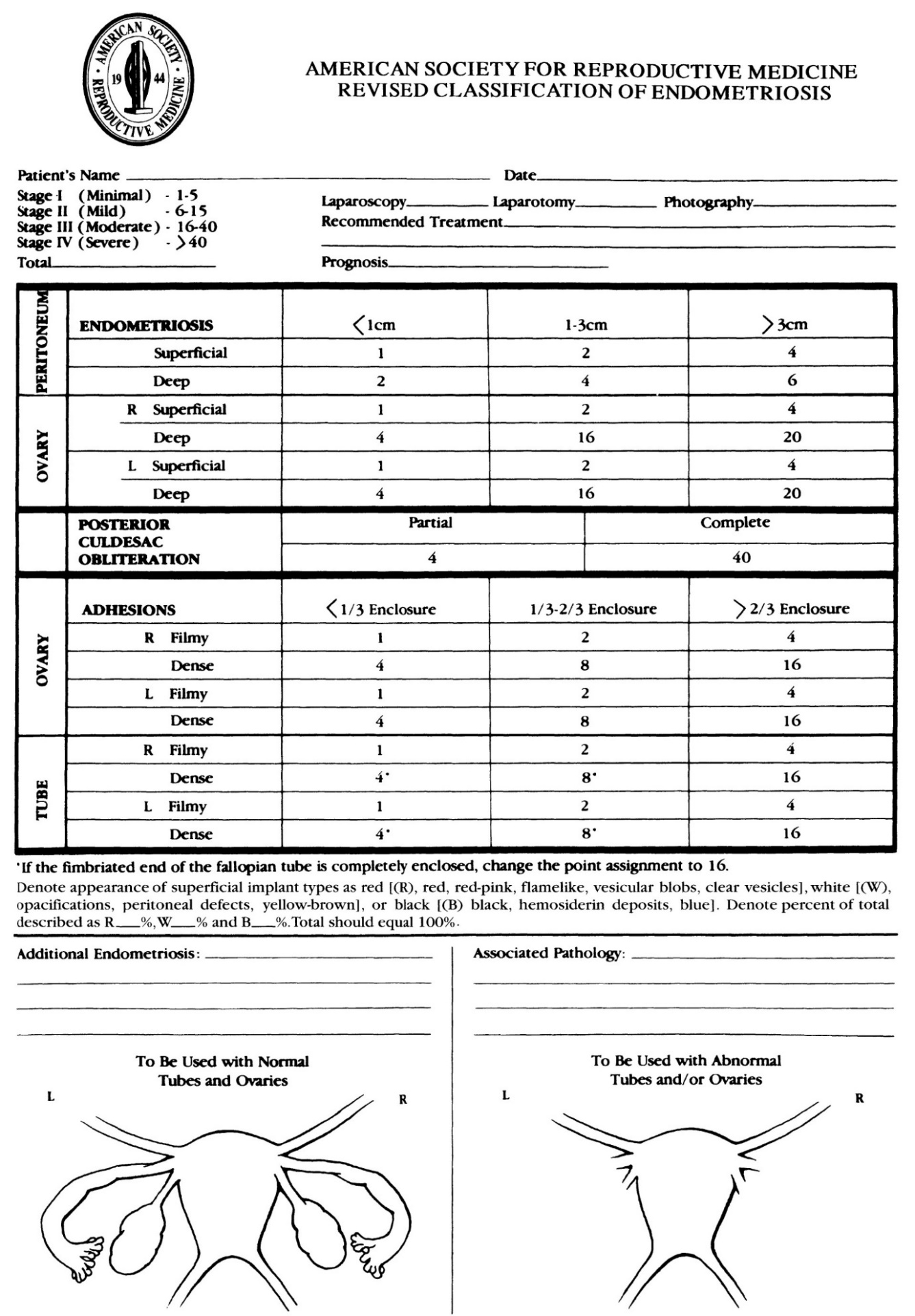


**Supplementary Figure S1** Revised American Society for Reproductive Medicine (rASRM) classification of endometriosis. Reprinted from the Revised American Society for Reproductive Medicine classification of endometriosis: 1996. *Fertil Steril* 1997; **67**: 817–821 (1997). Copyright © 1997 American Society for Reproductive Medicine, with permission from Elsevier. All rights reserved.


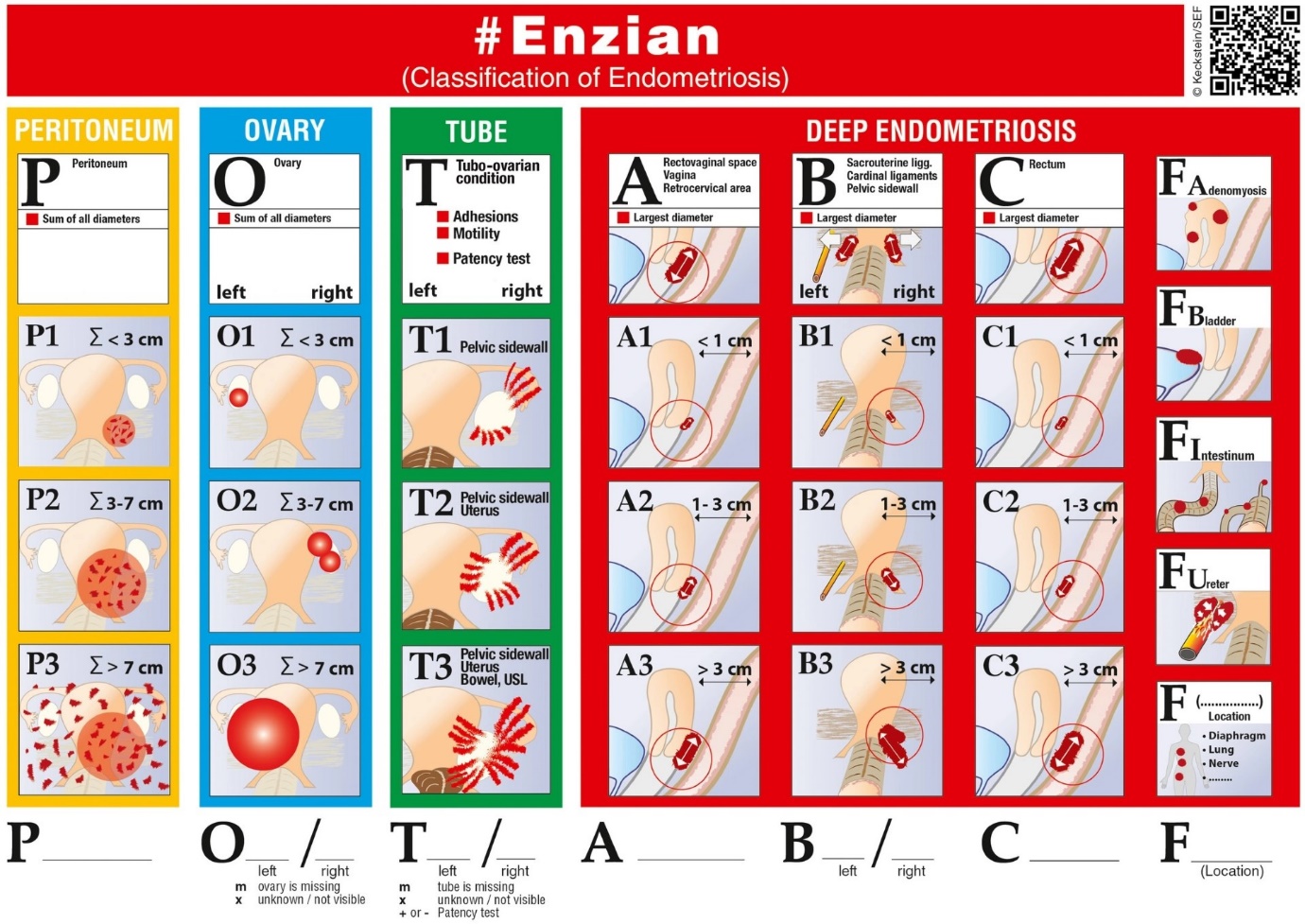


**Supplementary Figure S2** #Enzian classification system for women with superficial, ovarian and deep endometriosis. Reprinted from Keckstein *et al*. ^(Keckstein, Saridogan, Ulrich, Sillem, Oppelt, Schweppe, Krentel, Janschek, Exacoustos, Malzoni, Mueller, Roman, Condous, Forman, Jansen, Bokor, Simedrea and Hudelist, 2021)^, with permission from J. Keckstein. Copyright © 2021 The Authors. Published by John Wiley & Sons Ltd on behalf of Nordic Federation of Societies of Obstetrics and Gynecology (NFOG). Sacrouterine ligg/USL, uterosacral ligaments.


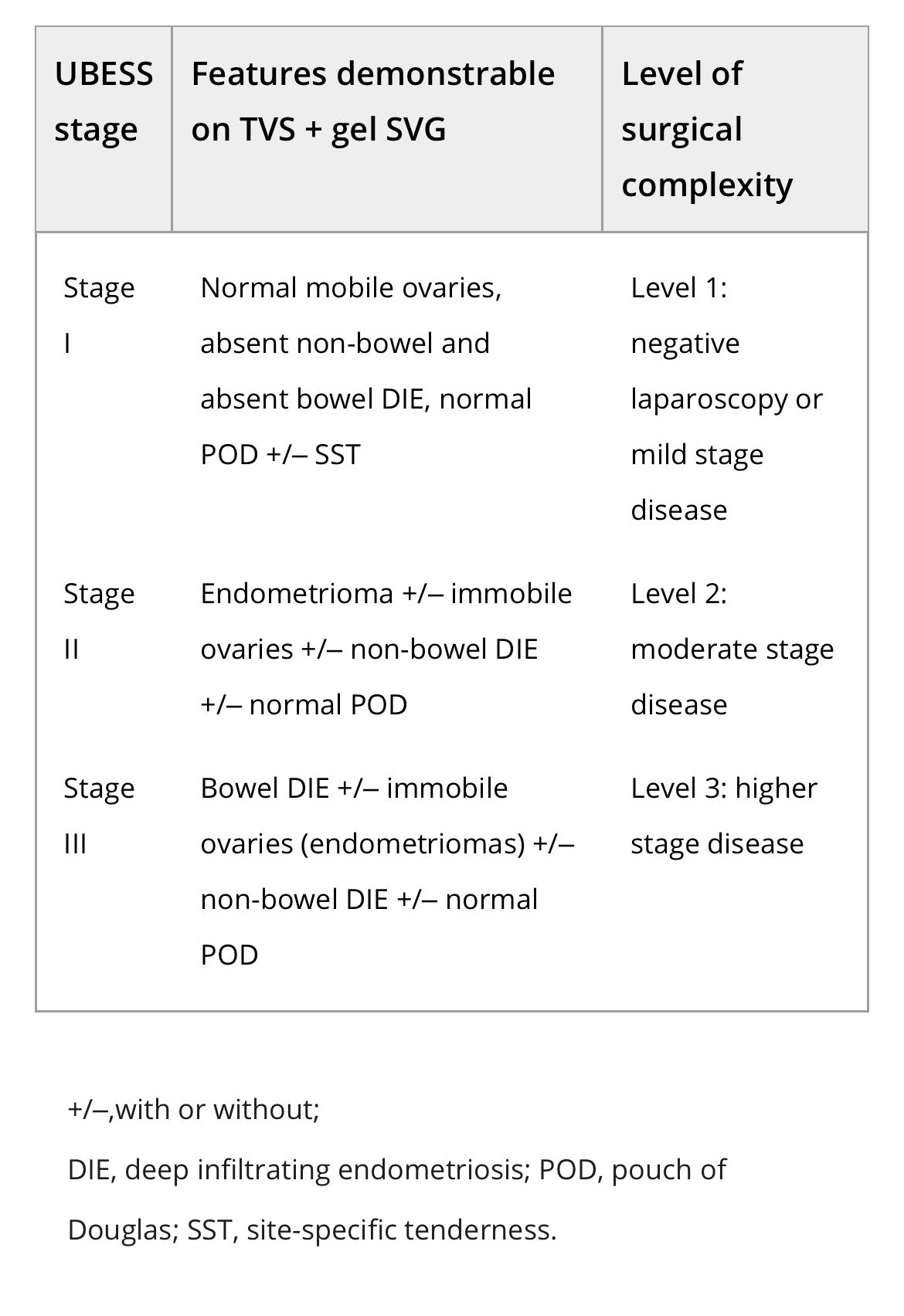


**Supplementary Figure S3** Ultrasound-based Endometriosis Staging System (UBESS), with sonographic features demonstrable on transvaginal ultrasound (TVS) and its prediction of level of surgical complexity. Adapted from Menakaya *et al*. (Menakaya, Reid, Lu, Gerges, Infante and Condous, 2016), with permission from ISUOG. SVG, sonovaginography.


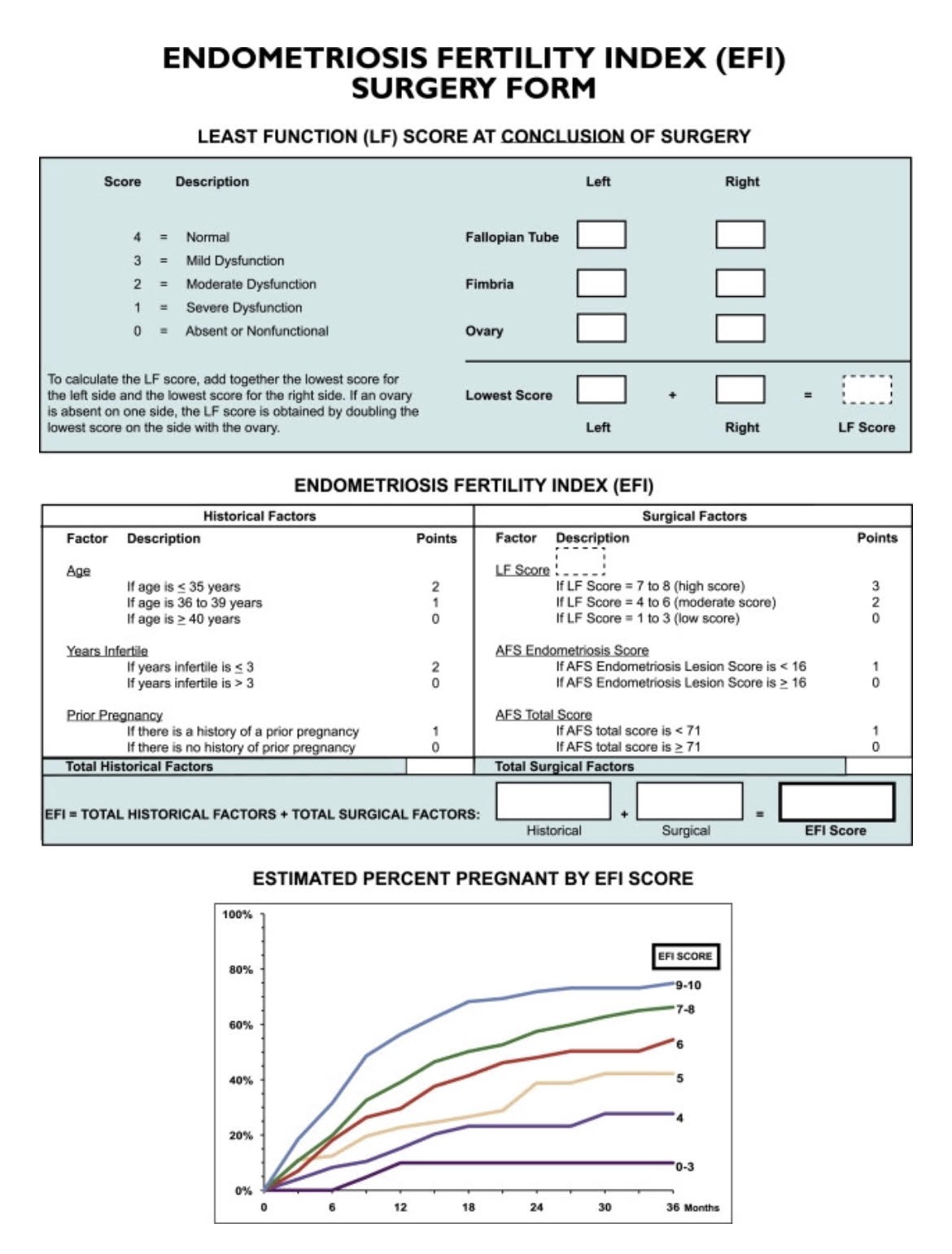


**Supplementary Figure S4** Endometriosis fertility index (EFI) system. This score predicts fertility outcome for women who attempt non-*in-vitro* fertilization conception following surgically documented endometriosis. Reprinted from Adamson GD, Pasta DJ. Endometriosis fertility index: the new, validated endometriosis staging system. *Fertil Steril* 2010; **94**: 1609–1615^7^. Copyright © 2010 American Society for Reproductive Medicine, with permission from Elsevier. All rights reserved. AFS, American Fertility Society.


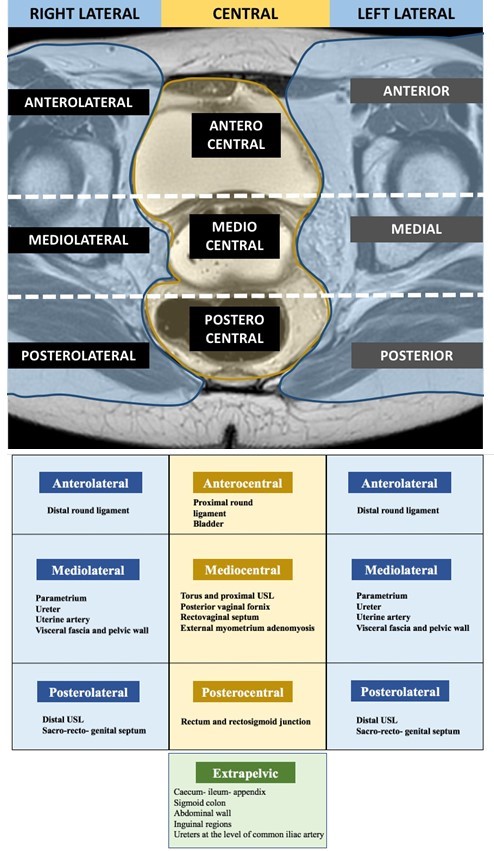


**Supplementary Figure S5** Magnetic resonance imaging (MRI) lexicon and deep pelvic endometriosis index (dPEI) classification: low extension (score 1 or 2), moderate extension (score 3 or 4) or severe extension (score 5 or more). Reproduced from Rousset P, Florin M, Bharwani N, Touboul C, Monroc M, Golfier F, Nougaret S, Thomassin-Naggara I, Group E. Deep pelvic infiltrating endometriosis: MRI consensus lexicon and compartment-based approach from the ENDOVALIRM group. *Diagn Interv Imaging* 2023; **104**: 95–112 ^(Rousset, Florin, Bharwani, Touboul, Monroc, Golfier, Nougaret, Thomassin-Naggara and Group, 2023)^. Copyright © 2022 The Author(s). Published by Elsevier Masson SAS on behalf of Société française de radiologie. All rights reserved.
