# Supplementary material for: Non-invasive imaging techniques for diagnosis of pelvic deep endometriosis and endometriosis classification systems: an International Consensus Statement,
Source: Hum Reprod Open. 2024 May 29;2024(3):hoae029. doi: 10.1093/hropen/hoae029 (PMC11134890; doi:10.1093/hropen/hoae029)
Supplement: hoae029_Supplementary_File_S1_S2 [file hoae029_supplementary_file_s1_s2.docx]

**Supplementary File S1** Search strategy used for identification of potentially relevant studies with corresponding number of studies found (literature search using MEDLINE).

1 deep.mp. 281 819

2 endometriosis.mp. or exp Endometriosis/ 30 750

3 1 and 2 2004

4 imaging.mp. 2 264 021

5 ultrasound.mp. 284 805

6 sonography.mp. 34 198

7 magnetic resonance.mp. 816 546

8 shift imaging.mp. 1092

9 exp Magnetic Resonance Imaging/ 503 906

10 proton spin.mp. 735

11 spin echo.mp. 15 398

12 MRI.mp. 287 756

13 NMR.mp. 191 443

14 exp Tomography, X-Ray Computed/ or computed tomography.mp. 612 390

15 computer assisted tomography.mp. 824

16 beam tomography.mp. 566

17 Computerized Axial Tomography.mp. 1339

18 CT.mp. 392 841

19 CAT.mp. 123 972

20 4 or 5 or 6 or 7 or 8 or 9 or 10 or 11 or 12 or 13 or 14 or 15 or 16

or 17 or 18 3 080 240

21 3 and 20 692

**Supplementary File S2** Levels of evidence and grades of statement used in this work (Guyatt et al., 2008, Howick et al., 2011).

**Oxford Centre for Evidence-Based Medicine (CEBM) Levels of Evidence**

**1a**: Systematic review (with homogeneity) of Level-1 diagnostic studies; or clinical decision rule with Level-1b studies from different clinical centers

**1b**: Validating cohort study with good reference standards; or clinical decision rule tested within one clinical center

**1c**: Absolute SpPins and SnNouts*

**2a**: Systematic review (with homogeneity) of Level > 2 diagnostic studies

**2b**: Exploratory cohort study with good reference standards; or clinical decision rule after derivation, or validated only on split-sample or databases

**3a:** Systematic review (with homogeneity) of studies Level ≥ 3b

**3b:** Non-consecutive study; or without consistently applied reference standards

**4:** Case–control study; poor or non-independent reference standard

**5:** Expert opinion without explicit critical appraisal, or based on physiology, bench research or ‘first principles’

**Grades of Statement**

**A (High):** Further research is very unlikely to change our confidence in the estimate of effect.

• Several high-quality studies with consistent results

• In special cases: one large, high-quality multicenter trial

**B (Moderate):** Further research is likely to have an important impact on our confidence in the estimate of effect and may change the estimate.

• One high-quality study

• Several studies with some limitations

**C (Low):** Further research is very likely to have an important impact on our confidence in the estimate of effect and is likely to change the estimate.

• One or more studies with severe limitations

**D (Very low):** Any estimate of effect is very uncertain.

• Expert opinion

• No direct research evidence

• One or more studies with very severe limitations

Note: A minus sign ‘–’ may be added to the level of evidence to denote evidence that fails to provide a conclusive answer because it is either: (a) a single result with a wide confidence interval; or (b) a systematic review with considerable heterogeneity. Such evidence is inconclusive, and therefore can only generate Grade-D recommendations. *‘Absolute SpPin’ is a diagnostic finding whose specificity is so high that a positive result rules in the diagnosis; ‘Absolute SnNout’ is a diagnostic finding whose sensitivity is so high that a negative result rules out the diagnosis.

**References**

Guyatt GH, Oxman AD, Vist GE, Kunz R, Falck-Ytter Y, Alonso-Coello P, Schünemann HJ; GRADE Working Group. GRADE: an emerging consensus on rating quality of evidence and strength of recommendations. *BMJ* 2008;**336**:924–926.

Howick J, Chalmers I, Glasziou P, Greenhalgh, T., Heneghan C, Liberati A, Moschetti I, Phillips B, and Thornton H. The 2011 Oxford CEBM Levels of Evidence (Introductory Document), Oxford Centre for Evidence-Based Medicine, 2011 <https://www.cebm.ox.ac.uk/resources/levels-of-evidence/ocebm-levels-of-evidence>.
